# Wearable-derived Sleep Measurements are Associated with Long-COVID in the RECOVER Adult Cohort

**DOI:** 10.21203/rs.3.rs-7422764/v1

**Published:** 2025-09-03

**Authors:** Sairam Parthasarathy, Shari Brosnahan, Solveig Sieberts, Elias Neto, Yanling Li, Meghasyam Tummalacherla, Heather-Elizabeth Brown, Sy-Miin Chow, Jessilyn Dunn, Monika Haack, Shekh Md Islam, Marissa Jacobs-Diggs, Yihang Jiang, Joe Kossowsky, Aric Prather, Nadia Raytselis, Nima Salimi, Mirna Ayache, Logan Bartram, Jacqueline Becker, Alicia Chung, James DelAlcazar, Valerie Flaherman, Kelly Gibson, Minjoung Go, Ramkiran Gouripeddi, Jenny Han, Mathew Hoffman, Sarah Jolley, J. Kelly, Ziad Koberssy, Jerry Krishnan, Adeyinka Laiyemo, Joyce Lee-Iannotti, Emily Levitan, Diego Mazzotti, Grace McComsey, Alem Mehari, Megumi Okomura, Thomas Patterson, Michael Peluso, Bharati Prasad, Orlando Quintero, A Ryerson, Prachi Singh, Upinder Singh, Monica Verduzco-Gutierrez, Peter Whitesell, Natasha Williams, Juan Wisnivesky, Janet Mullington, Susan Redline, Elizabeth Karlson

**Affiliations:** University of Arizona; Division of Pulmonary and Critical Care Medicine, New York University Grossman School of Medicine, NYU Langone Health; Sage Bionetworks; Sage Bionetworks; Pennsylvannia State University; Sage Bionetworks; National Community Engagement Group; The Pennsylvania State University; Duke University; Beth Israel Deaconness Medical Center; Duke University; Family Advisory Coucil, New York Presbyterian Morgan Stanley Children’s Hospital; Duke University; Boston Children’s Hospital; University of California San Francisco; Emory University; Sage Bionetworks; Case Western Reserve University; Icahn School of Medicine at Mount Sinai; Icahn School of Medicine at Mount Sinai; NYU Grossman School of Medicine; Providence Swedish Medical Center; University of California San Francisco; Metrohealth System; Stanford University; University of Utah; Emory University; University of Utah; University of Colorado AMC; University of California, San Francisco; Case Western Reserve University; Division of Pulmonary, Critical Care, Sleep and Allergy, University of Illinois at Chicago; Howard University College of Medicine; Barrow Neurological Institute; University of Alabama at Birmingham; University of Kansas Medical Center; Case Western Reserve University; Howard University; University of California San Francisco; University of Texas Health Science Center at San Antonio; Division of HIV, Infectious Diseases, and Global Medicine, University of California San Francisco, San Francisco, CA USA; University of Illinois; Stanford University; Kaiser Permanante Georgia; Pennington Biomedical Research Center; Stanford University; University of Texas San Antonio; Howard University; New York University; Icahn School of Medicine at Mount Sinai; Beth Israel Deaconess Medical Center; Brigham and Women’s Hospital; Brigham and Women’s Hospital,

**Keywords:** PASC, Long COVID, Wearables, Sleep, Digital Health, Heart Rate Variability

## Abstract

Wearables yield a wide array of sleep-related measures that are relevant to Long COVID. We leveraged wearables-derived sleep measures (WDSM) to identify differences between individuals with Long COVID (LC) versus individuals with possible or no LC in the RECOVER adult cohort. We found significant associations between LC and reduced heart rate variability measured during sleep and increased nightly variability in sleep duration after adjusting for confounders. Moreover, LC was independently associated with lower sleep efficiency, greater variability of nighttime sleep timing, higher resting heart rate, lower respiratory rate during rapid eye movement (REM) sleep, prolonged REM sleep onset latency, worse global physical and mental health. Cluster analysis identified distinct multidimensional patterns of WDSM that are associated with LC and quality of life. Together, the strong association between WDSM, or WDSM clusters, with LC provides a potential biomarker for future validation efforts to detect LC and monitor treatment effectiveness.

## Introduction

Long COVID (LC) is estimated to affect 6.9% of the U.S. population and costs an estimated $3.7 trillion over 5-years and continues to compound the health and economic burden of the nation^1–4^. However, there is marked under-detection of LC and one of the prominent reasons includes the lack of a clear diagnostic test^5,6^. In fact, little correlation between standard clinical laboratory tests and patient reported symptoms of LC has been observed^7^. Conceivably, diagnostic tests fail to demonstrate consistent associations with LC due to the heterogeneity of this condition that involves multiple organ systems and multiple pathobiological processes^8,9^.

Sleep-related symptoms are the third most common symptom of LC and in some reports nearly 40% of patients with LC report sleep disturbances^10,11^. Moreover, sleep, like LC, is interconnected with multiple organ systems and biological processes such as cognition, immune regulation, and anti-inflammatory processes which are dysregulated in individuals with LC^12–14^. This is further supported by evidence suggesting that preexisting sleep issues may play a role in the development of LC^15–17^. Sleep can be readily measured by wearables^18–21^. Considering the cross-cutting and interconnected nature of sleep and LC, it is conceivable that wearables-derived sleep measures (WDSM; Table 1) may provide a digital indicator of LC. WDSM could further our understanding of the relationship between LC and sleep by enabling longitudinal collection in sufficiently powered cohorts. Accordingly, we investigated whether WDSM could identify differences between individuals with a high burden of LC symptoms versus those that had low or no burden of LC symptoms. As a secondary objective we investigated whether there are differences in WDSM for participants with and without individual symptoms of LC. Finally, we conducted cluster analysis of WDSM to identify multidimensional patterns of sleep changes that are associated with LC and health-related quality of life.

**Figure 1:**
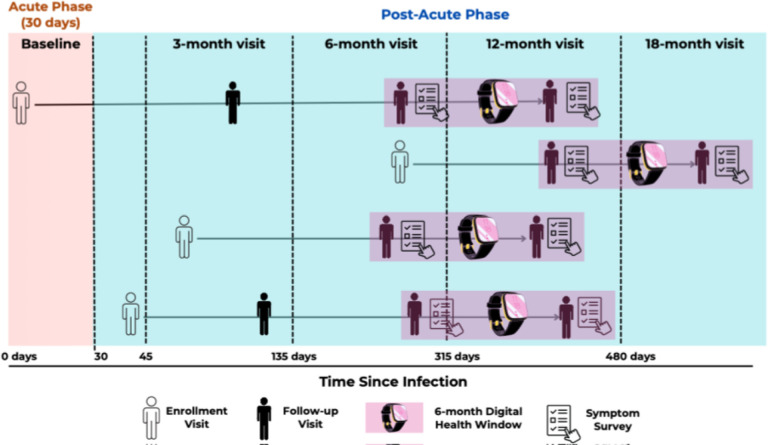
RECOVER participants were invited to enroll in the Digital Health Program starting in March 2023. For this analysis, we used the first 6-month window of digital health data that was at least 6-months post-infection and compared it to 2 consecutive symptom surveys.

To do so, we leveraged the WDSM in adult participants in the RECOVER Adult Cohort Study who were also enrolled in the RECOVER Digital Health Platform: A total of 1,230 individuals with WDSM over 6 months (minimum of 5 days of valid measurements) for a total of 151,045 nights of measurements (Fig. 1). The WDSM were complemented by two symptom-surveys over a 6-month period in the RECOVER Adult Observational cohort that yielded information regarding LC status based on the 2024 RECOVER Long COVID Research Index (LCRI) that classifies highly symptomatic LC (“likely LC”) as Long COVID Research Score ≥ 11 as a weighted index of LC symptoms.^22^ WDSM includes sleep time, as well as metrics of sleep quality and sleep stages that are estimated by monitoring heart rate variability (HRV), the variation in time intervals between heartbeats, movement patterns and oxygen saturation.^20,23^ We analyzed 14 Wearables-derived variables in relation to the LC status (any LCRI score ≥ 11 vs LCRI score < 11 at all timepoints) measured over 6-months to test the robustness of the observed associations. Although some smaller studies have assessed some of the wearables-derived data in patients with Long COVID^24,25^ they have largely focused on activity-related measures. A comprehensive assessment of WDSMs in relation to well-defined LC status has not yet been performed^24,26^. Association between WDSM and LC, if present, has wide generalizability and a patient-powered approach for potentially detecting and monitoring the time-based or treatment-driven trajectories of LC.

## Results

### Cohort demographics

Among the 1,262 sleep digital health participants, 433 were classified as Likely LC (LCRS ≥ 11) for at least one of the two timepoints, 529 had lower symptom burden at both timepoints (possible LC; 11 > LCRS > 0), and 299 had no LC symptoms (LCRS = 0) at either timepoint (Table 2). Female participants were more likely to be in the LC group (81% female in likely LC and 74% in both the possible LC and no LC symptoms group). White, non-Hispanic participants were more likely to be in the LC group (70%, 68% and 63% for the likely, possible and no LC groups, respectively). In contrast, Asian, non-Hispanic participants were less likely to be in the LC likely group (3%, 5.9% and 11% for the likely, possible and no LC symptoms groups, respectively). The median age at enrollment was similar across groups: 46 years (IQR: 37–56) for the likely LC group, 44 years (IQR: 34–59) for the possible LC group, and 43 years (IQR: 28–58) for the no LC group.

### Association between Long COVID and Sleep

**Figure 2:**
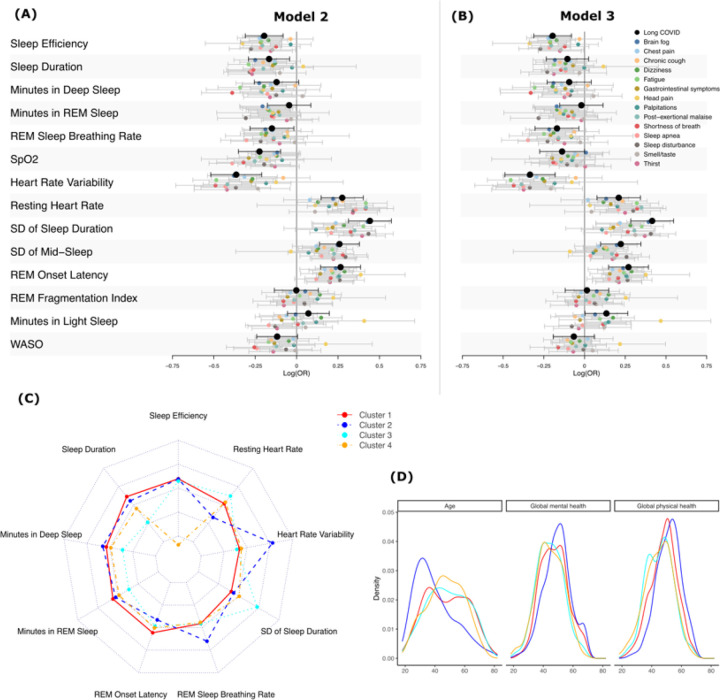
Effect sizes for WDSM associations with LCRI status (black) and individual symptoms of LCRI and LC adjusting for **(A)** age, sex and race/ethnicity (model 2) or **(B)** age, sex, race/ethnicity and BMI. **(C)** Hierarchical clustering of WDSM result in four clusters. The clusters are characterized by good sleep and average resting heart rate and HRV (cluster 1), low resting HR and high HRV and REM breathing rate (cluster 2), low sleep time and high variability (cluster 3) and low sleep efficiency (cluster 4). **(D)** The distribution of age, PROMIS Global Mental and Global Physical Health Scores.

We examined the association between LCRI group and the digital sleep measures using two primary models: age, sex and race/ethnicity (model 2) and age, sex, race/ethnicity and BMI (model 3). We additionally examined an unadjusted model (model 1) and a full model which adjusted for age, sex, race/ethnicity, BMI, alcohol consumption, smoking, seasonality and two data quality metrics (see Methods). The results for these models were qualitatively similar to those for models 2 and 3, respectively. Under model 2 (age, sex and race/ethnicity), likely LC was associated with decreases in sleep efficiency, sleep duration, REM sleep breathing rate, SpO2, HRV, and increases in resting HR, SD of sleep duration, SD of mid-Sleep, and REM onset latency (corrected p-value ≤ 0.05) Table 3; **Table S2; Figure S1**; Fig. 2(A)). The associations of LCRI with sleep duration and SpO2 become non-significant (adjusted p-value = 0.160 and 0.077, respectively) when further including BMI in the model (model 3), implying that these effects may be at least partially explained by BMI (**Table S3; Figure S1;** Fig. 2(B), **Figure S1**). The association of SD of sleep duration and SD of mid-sleep suggest that participants with likely LC show more between-night variability in sleep (or irregular sleep) patterns. A sensitivity analysis with and without weekend days showed similar results (**Figure S5**) suggesting that these effects are not driven by social jetlag differences (i.e., differences in sleep timing between weekday and weekends) but instead represent a broader pattern of sleep irregularity. A sensitivity analysis examined the effect of increasing the minimum available data to 14 days (versus 5 in the primary analyses). The results were qualitatively similar to the primary analysis (**Figures S3 & S4**).

### Associations among LC Symptoms and Sleep

We also examined the LC symptoms associated with the WDSM. The measures included were: brain fog, chest pain, chronic cough, dizziness, fatigue, gastrointestinal (GI) symptoms, head pain, palpitations, post exertional malaise (PEM), shortness of breath (SOB), sleep apnea, sleep disturbance, loss of smell and/or taste, and thirst as described earlier^22,27^. In models 2 and 3, all LC symptoms were associated with at least one sleep metric: brain fog (9), sleep disturbance (9), dizziness (8), fatigue (8), PEM (7), SOB (6), GI symptoms (7), thirst (6), sleep apnea (5), head pain (3), loss of smell or taste (3), chest pain (3), chronic cough (3), and palpitations (3) (Fig. 2A, 2B, S9, S10, S12-S25). Overall, decreased sleep efficiency and HRV, as well as increased SD of sleep duration and longer REM onset latency were associated with almost all symptoms examined (Fig. 2A, 2B; S9, S10, S12-S25). Complete tables by symptom and each model can be found in **tables S9-S64.**

Increased brain fog was associated with a decrease in sleep efficiency, sleep duration, minutes in deep sleep, minutes in REM sleep, REM sleep breathing rate, HRV and an increase in SD of sleep duration, SD of mid-sleep, and REM onset latency. Increased resting HR was significant in model 2 but lost significance with the addition of BMI perhaps as higher BMI is associated with higher resting HR (Fig. 2A, 2B, S15; **Tables S22, S23**).

Dizziness was associated with a decrease in sleep efficiency, minutes in deep sleep, minutes in REM sleep, REM sleep breathing rate, HRV and increase in SD of sleep duration, REM onset latency and minutes in light sleep. For dizziness the associations of several WDSM increased with subsequent regression models (**Figure S20; Table S41-S44**).

Self-reported sleep disturbance was associated with a decrease in sleep efficiency, sleep duration, minutes in REM, REM sleep breathing rate, and HRV, and an increase in resting HR, SD of sleep duration, SD of mid-sleep, and REM onset latency (**Tables S61-S64**).

Fatigue was associated with a decrease in sleep efficacy, sleep duration, minutes in deep sleep, REM sleep breathing rate, HRV and in increased resting HR, SD of sleep duration, and REM onset latency. Similar to brain fog, in both sleep disturbance and fatigue, SpO2 does not retain its significance between model 2 and 3 related to BMI correction, while the REM sleep breathing rate association strengthens in significance with the inclusion of additional covariates (**Table S37-S40)**.

### Sleep and health-related quality of life

We also examined the association between the WDSMs and two measures of quality of life (QoL) from the Patient-Reported Outcome Measurement Information System (PROMIS) Global Health scale, the Global Physical Health (GPH) index T-scores (4 items on overall physical health, physical function, pain, and fatigue) and Global Mental Health (GMH) T-scores (4 items on quality of life, mental health, satisfaction with social activities, and emotional problems).^28^ For each index, better QoL was associated with better sleep efficiency, increased sleep duration, more time in deep sleep, higher HRV, a lower resting HR, more sleep regularity (lower SD of sleep duration and SD of mid-sleep), and shorter REM onset latency. Wake after sleep onset (WASO) and SpO2 were significant in model 2 but did not retain significance when controlled for BMI in both scores as well. Increased time in REM sleep was associated with GPH but not GMH (Fig. 2A, 2B; **Figures S26 & S27; Tables S65-S72**).

### Cluster analysis of digital sleep measures

To understand participant patterns with respect to the WDSM, we performed hierarchical clustering based on a subset of digital sleep measures (sleep efficiency, sleep duration, minutes in deep sleep, minutes in REM sleep, REM onset latency, REM sleep breathing rate, SD of sleep duration, HRV and resting HR) (Fig. 2(C); **Table S73)**. These were chosen as the most consistently correlated with likely LC and presence of LC symptoms. Visual inspection of the scree plot (**Figure S28**) identified four primary clusters (Fig. 2(C)). Cluster 1 consists of participants manifesting better cardiovascular fitness with a lower baseline heart rate (mean(SD) = 67.94(7.28)), but lower HRV (mean(SD) = 25.54(10.19)), with an increased REM Breathing Rate (mean(SD) = 14.18(1.70)). Sleep efficiency, duration and REM Onset Latency were high (mean(SD) = 94.16(1.9), 381.79(48.74) and 124.25(34.91), respectively), and SD of sleep duration was low (mean(SD) = 93.11 (29.09)). Cluster 2 represents a group with higher efficiency, longer sleep duration and lower variability (mean(SD) = 94.00(2.30), 366.12(55.66) and 97.62(31.8), respectively). Participants in this cluster displayed an increase both in minutes in REM sleep (mean(SD) = 85.13 (20.02)) and a decrease in REM onset latency (mean(SD) = 106.82 (24.93)). They showed the highest HRV (mean(SD) = 52.04 (22.60)) and lowest resting HR (mean(SD) = 61.74(7.15)). The cluster 3 group was characterized by a short sleep time (mean(SD) = 287.10(58.26)), reduced minutes in both deep and REM sleep (mean(SD) = 54.19(15.96) and 71.72(17.53), respectively), and greater SD of sleep duration (mean(SD) = 143.73(43.11)), as well as a higher resting HR (71.36(8.25)). Cluster 4 shares the feature of sleep duration < 6h with cluster 3 but is further characterized by low sleep efficiency (mean(SD) = 63.36(9.68)) but moderate sleep duration (mean(SD) = 337.73(75.04)).

Cluster 4 showed the highest proportion of LC positive participants (48%; Table 4). It also showed lower GPH and GMH scores (median(IQR) = 45 (39–51) and 45 (39–52), for Physical and Mental, respectively) (Fig. 2(D)). It also showed a higher proportion of females (84% vs 79%, 72% and 73% for clusters 1, 2, and 3, respectively). In contrast, cluster 2, showed the lowest proportion of likely LC positive participants (20%) with high GPH (median(IQR) = 52 (46–58)) and GMH (median(IQR) = 50 (44–55)). This cluster had younger participants than the other three (median age = 36 vs 48, 49, and 47 for clusters 1, 3 and 4, respectively) (Fig. 2(D)). Clusters 1 and 3 showed a moderate proportion of likely LC participants (35% and 41%, respectively). Cluster 1 showed higher GPH scores (median(IQR) = 49 (42–54) versus 46 (39–52) for cluster 3). It also had a higher proportion of female participants (79% versus 73%).

### Profile Analysis

In order to further understand the patterns of WDSMs associated with participant self-reports of sleep apnea and sleep disturbance, as well as self-reported quality of life measures (i.e. PROMIS GPH and GMH Indices), we employed Criterion Profile Analysis (CPA)^29^. Sleep apnea was associated with a decrease in REM sleep breathing rate and increase of variability of sleep duration, while sleep disturbance was associated with both those factors, as well as a decrease in sleep efficiency, minutes in REM sleep and an increase in resting HR (**Figure S29**). Lower scores for the GPH and GMH measures were associated with decreases in sleep efficiency and minutes in deep sleep, as well as lower HRV (**Figure S30**). Lower scores are also associated with increases in REM onset latency and SD of sleep duration. Additionally, an increase in resting HR was associated with lower GPH but did not reach significance for a change of GMH. Likewise, a lower REM sleep breathing rate was associated with lower GMH but did not reach significance for a change of GPH. By comparing the absolute values of CPA scores, we found that variability of sleep duration generally played a more important role than other digital sleep measures in predicting sleep problems and physical/mental health. Significant pattern effects were found for all outcome variables (sleep apnea: F-statistic(17, 1085) = 7.69, p < 0.001; sleep disturbance: F-statistic(17, 1085) = 3.48, p < 0.001; GPH: F-statistic(17, 1100) = 15.30, p < 0.001; GMH: F-statistic(17, 1100) = 9.14, p < 0.001).

### Stratified Analyses

We performed stratified analyses by age (< 45, 45 to 65 and > 65) (**Figure S31**), sex (**Figure S32**) and race and ethnicity (**Figure S33**). With respect to age, the associations and directions of effect were similar for most of the variables which were significant in the main analysis (i.e. sleep efficiency, HRV, resting HR, SD of sleep duration, SD of mid-sleep, REM onset latency) (**Figure S31**). Even though some associations showed discordant directions none reached statistical significance, with none of the associations reaching statistical significance in the older group (over 65). Given the reduced sample sizes in some of these age groups (n = 611, 474, and 145 for the < 45, 45–65, and > 65 age groups, respectively), some reduction in power was to be expected. Interestingly, we did observe a significant positive association between likely LC and minutes spent in light sleep in the youngest subgroup, which was not observed in either subgroup or the combined analyses but may be due to reduced power.

The patterns of change in WDSM with likely LC were generally similar across both sexes (**Figure S32**). However, the magnitudes and statistical significance did vary across the sex groups. Both male and females with LC showed significant decrease in HRV, increased in resting HR, SD sleep duration and SD of mid-sleep, while only females showed a significant decrease in sleep efficiency, SpO2 and increase in REM onset latency which was not seen as significant in the males. Likewise, males had a significant decrease in sleep duration and WASO that was not observed in women.

## Discussion

In this work involving the largest cohort of wearables measurement in individuals with LC, we found biologically plausible differences in WDSM between individuals with likely LC and those with low or no LC symptom burden over a six-month measurement period. Specifically, the strongest association was observed for reduced HRV measured during sleep and increased variability in sleep duration. HRV measures the beat-by-beat variability that reflects the dynamic interaction between sympathetic and parasympathetic tone within the autonomic nervous system and reflects overall health with higher HRV associated with cardiovascular health and better response to stress. There are numerous prior reports of reduced HRV – measured by conventional electrocardiogram – in individuals with LC that could exemplify the underlying autonomic instability that may be triggered or exacerbated by a COVID infection or underlie and/or drive LC presentation and/or symptoms as dysautonomia, Postural Orthostatic Tachycardia Syndrome (POTS), and PEM^30,31^. While many studies have previously used electrocardiograms, Holter monitors, and cardiac belts, the photoplethysmography-based wearables used in our study to yield reliable HRV data poses a promising alternative and practical approach^32^. Such opportunity has been capitalized by others to demonstrate a reduction in HRV during acute SARS-CoV-2 infection but, to our knowledge, there are no prior wearables-based studies demonstrating the association between reduced HRV and LC^33^. The observed association between wearables-derived reduced HRV during sleep and LC further supports autonomic imbalance as one of the mechanistic underpinnings of LC. Additionally, it lends itself for longitudinal monitoring of patients undergoing treatments that targets the autonomic imbalance such as slow-paced breathing.^34^ While many studies have previously used electrocardiograms, Holter monitors, and cardiac belts, the photoplethysmography-based wearables used in our study to yield reliable HRV data during sleep poses a promising alternative and practical approach and considering the relative immobility may provide a more accurate measure and minimize artifacts induced by activity^21,32^.

We also find a strong association of LC with SD of sleep duration and to a lesser degree SD of mid-sleep, measures of sleep irregularity. Irregular sleep duration and timing (markers for circadian misalignment and its myriad effects of healthy physiology) have recently been identified to be associated with insulin resistance and dyslipidemia and to predict incident cardiovascular disease, cognitive decline, and all-cause mortality^35,36^. In the context of a multi-dimensional model for sleep health, increased irregularity in sleep-wake patterns has been shown to cluster with other sleep dimensions, including insufficient sleep duration, poor sleep efficiency, and sleep-disordered breathing^37^. Improving sleep regularity may be an important strategy for more broadly improving sleep health, and in fact is a core principle for sleep hygiene interventions. Interestingly, recent reports indicate increased prevalence of cardiovascular events among individuals hospitalized for COVID-19 or even in mild cases of SARS-CoV2 infection when associated with certain ABO blood types^38^. Coehlo et al have previously described marked instability in polysomnographically measured sleep patterns with both long and short bouts of objectively measured sleep in individuals with LC^39^. An ongoing RECOVER clinical trial in fact is targeting sleep regularity as a strategy for improving LC-related sleep disturbance. The observed variability in sleep duration and mid-sleep (timing of nighttime sleep) in our study may serve as a biomarker for further stratification of individuals with greater likelihood of adverse long-term outcomes of LC and presents additional opportunity to augment current risk scores for predicting cardiovascular events in these individuals^38^.

We observed that LC was also associated with lower sleep efficiency and a tendency for reduced sleep duration. This contrasts with a prior report of similar wearables-measured sleep quantity between individuals with LC and those without LC following a known SARS-CoV-2 infection^24^. In this prior study by Radin et al, participants with LC (n = 279) and without LC (n = 274) were followed up to one year but sleep measures besides sleep quality were unfortunately not reported^24^. Conceivably, better case identification using the LCRI and larger sample size of our study (n = 1,230 including 417 likely LC cases) may have resulted in greater power to observe statistical differences, including lower sleep efficiency and tendency for reduced sleep duration in participants with persistent LC compared to those without LC. Similar to the findings in our study, Radin et al found more variable sleep duration and higher resting HR in LC which may signify sleep fragmentation, reduced parasympathetic tone, possibly in response to nocturnal hypoxia and sleep apnea. In our study, reflectance oximetry levels (SpO2) measured by the wearables were lower in individuals with likely LC when compared to those with low or no LC symptoms. Nocturnal hypoxia has been previously described in individuals with LC and may reflect either the greater prevalence of obstructive sleep apnea (OSA) in these individuals with LC, sleep-related hypoventilation, or underlying pulmonary complications of LC^15,40–42^. Interestingly, nocturnal hypoxemia can be relatively asymptomatic in acute COVID due to posited alterations in chemosensitivity and control of breathing, but importantly may contribute to delayed detection that could be mitigated by wearables^40^. Such a phenomenon of altered chemosensitivity may persist in individuals with LC but this is unclear^40^. Obesity may further contribute to sleep-related hypoventilation and consequently lower oxygen saturation, which, in turn may contribute to fatigue and cognitive problems^43^. In our study, after adjustment for body mass index (BMI), the association between lower SpO2 and LC was lost suggesting that BMI (and comorbid OSA and sleep-related hypoxia or restrictive pulmonary phenotype) may explain the observed association between oxygen levels and LC^44–46^. Additionally, our observations of a lower respiratory rate during REM sleep further strengthens the possibility of comorbid OSA and sleep-related hypoxia playing a role in the LC pathophysiology^47^.

Prolonged REM sleep onset latency in individuals with LC has been previously described^48^. The reasons for delayed REM sleep latency are uncertain, but may be due to comorbid OSA and/or medications (e.g., Low Dose Naltrexone, sedative-hypnotics such as zolpidem, and SSRI/SNRI antidepressants prescribed for their “purported” protective effects against LC) that may suppress REM sleep^49^. Prolonged REM sleep latency has been associated with cognitive dysfunction and biomarkers of Alzheimer’s disease^50^.

In our study, WDSMs were strongly associated with worse GPH and GMH QoL ratings. Specifically, worse Mental and worse Physical scores were associated with multiple metrics of poor sleep including reduced sleep efficiency, reduced REM related breathing rate, reduced HRV during sleep, increased variability of mid-point sleep and sleep duration, prolonged REM latency, and higher resting HR during sleep. Importantly, such analysis of health-related quality of life measures, individual LC symptoms, and WDSM revealed marked internal consistency and external validity of the directionality of associations. The identification of associations of LC with multiple metrics of sleep is consistent with modern frameworks of sleep health that posit that sleep is a multidimensional construct composed of various interacting features of sleep that together better describe sleep health than individual metrics. The strongest associations between individual symptoms of LC and WDSM were observed for the most common and burdensome LC symptoms -- PEM, brain fog, fatigue, and dizziness.

Cluster analysis revealed four distinct patterns indicative of various manifestations of sleep disorder symptoms. A cluster (**cluster 3;** Fig. 2b) with clear reduction in sleep duration, increased resting HR, increased variability of sleep duration and reduced time in REM and deep sleep may signify a phenotype characterized by increased arousal and shorter, lighter, and more variable sleep, which may reflect LC-associated “insomnia phenotype”. Whereas, another cluster (cluster 2) is delineated by higher HRV, higher REM breathing rate (i.e., less apnea in REM), and lower resting HR and appears to denote a more “normal” phenotype and likely consisted of a more resilient younger age group of participants (Fig. 2b).

While the findings represent the cross-cutting nature of sleep and sleep-related physiological changes (heart rate, oxygen saturation), these findings may also denote the bidirectional association between LC and sleep. Taken together, the strong association between WDSMs with LC status and symptoms support their utility as a possible digital biomarker that may be further validated for identifying subgroups of patients with the LC condition who may experience different health trajectories and may benefit from targeted interventions, as well as for monitoring disease course and treatment effectiveness. The findings of lower oxygen saturation levels, lower HRV, and variability in sleep duration and timing with LCRI status provide potential targets for future interventions using positive airway pressure therapy or supplemental oxygen, slow paced breathing/vagal stimulation, and behavioral and circadian (e.g., light) approaches and the very same wearables can assist with monitoring the corresponding treatment response. Finally, identifying clusters of sleep-related metrics that reflect diverse domains of sleep health supports both the importance of applying a multi-dimensional sleep health framework in patients with LC, and suggests that this framework include measures of rest/sleep HRV, sleep variability, sleep efficiency, sleep duration, and metrics related to breathing and oxygenation during sleep.

## Methods

Institutional review boards at NYU Grossman School of Medicine, serving as a single Institutional Review Board, and other participating institutions reviewed and approved the protocol. All participants provided written informed consent to participate in research.

### Study Design

In RECOVER adult study design, participants, aged 18 years or older, were recruited at 83 sites in 33 states plus Washington, DC, and Puerto Rico, regardless of prior infection with SARS-CoV-2^51^. All participants completed a baseline set of surveys, a focused physical examination, and standard laboratory test sample collection at enrollment. Participants were followed prospectively with survey completion every 3 months and laboratory sample collection at enrollment and 6, 12, 24, 36, and 48 months after infection, or in the case of uninfected controls, the date of a negative test result (index date; Fig. 2). Subjects could enter the study at different times in relation to their initial COVID infection and were defined into cohorts including the acute infected (enrolled during first infection), post-acute (enrolled after initial COVID infection) and uninfected cohort (who enrolled prior to first infection but could be included in this study group if they were infected after study enrollment).^51^

### Participants

The Digital Health Program (DHP) is a component of RECOVER that was launched in 2023 to promote participant engagement and retention, and to collect new information that compliments and augments other RECOVER data including the collection of consumer-grade wearable “wrist-worn” device data (activity, heart rate, and sleep). All RECOVER Adult Cohort participants were invited to sign-up for DHP within an app that they downloaded to their device. They were asked to connect and share data from their own (bring your own (BYO) wearable devices (Fitbit, Apple Watch, Garmin, etc.) or order a free study device (Fitbit smartwatch, Sense 2 or Fitbit tracker, Charge 5).

As of November 11, 2024, a total of 6,497 RECOVER Adult participants enrolled in DHP, of which 4,104 (63%) connected a wearable device. Comparable wearables-derived sleep measures were only transmitted in Fitbit monitors which accounted for 2,696 (66%) subjects. Of those previously infected participants with Fitbit sleep data, 1,262 (47%) had at least 5 nights of wearables-derived sleep measures concurrent with symptom surveys during the 6-month data collection period and were included in the cohort analysis. An additional 474 (18%) participants had sufficient data but were withheld for future validation of findings.

### Exposures and Outcomes

Likely LC was defined by a Long COVID Research Index (LCRI) Score ≥ 11 at any point in time over a 6-month period post-infection. Possible LC was defined as LCRI score between 1–10, and the no LCRI symptom group had a LCRI score = 0^22^. The scores were based on the RECOVER data-derived LCRI from 2024 that used Lasso models to discriminate between SARS-CoV2 infected and non-infected participants in the RECOVER Adult Cohort (Geng 2024). Possible LC and no LC was collapsed into an LC indeterminate group^22^. Further details for defining LC status are provided in the statistical analysis section (see below). Sensitivity analysis was conducted using the LCRI Score as a continuous variable and referred to the LCRS.

LC symptoms were included based on items in the scoring system for either the RECOVER LCRI (2024) or the 2024 NASEM LC Common Symptoms Definition^8,22,27^. LC outcome variables included: Long COVID status, Brain Fog, Chest Pain, Chronic Cough, Dizziness, Fatigue, Gastrointestinal (GI) Symptoms, Head Pain, Palpitations, Post Exertional Malaise (PEM), Shortness of Breath (SOB), Sleep Apnea, Sleep Disturbance, Loss of Smell and/or Taste, and Thirst. Measures of quality of life included PROMIS GMH (the sum value of the responses to items Global02, Global04, Global05, and Global10r) and PROMIS GPH (sum value of the responses to items Global03, Global06, Global07r, and Global08r) of the PROMIS Scale v1.2.^28^

### Wearables-Derived Sleep Measures (WDSM)

We selected and summarized a set of Fitbit sleep measures and measures collected during sleep and summarized them over the six-month window. A summary of these sleep metrics, mapped to domains of sleep health, is provided in Table 1. This includes the mean of the following measures (with units): sleep efficiency (percent), total sleep duration (minutes), minutes in sleep stages (deep sleep, REM sleep, light sleep), WASO, REM onset latency (minutes), REM fragmentation index,^52^ resting heart rate (RHR, beats per minute during sleep and rest), heart rate variability (HRV) during sleep (ms), REM sleep breathing rate (breaths per minute), average oxygen saturation (SpO2; percent). We also examined across-night sleep variability metrics by including the within-individual standard deviation (SD) of sleep duration (minutes) and within-individual SD of sleep midpoint (SD of mid-sleep; minutes). For the standard deviation metrics, we performed a sensitivity analysis excluding weekend days, to examine the degree sleep variability was driven by weekday/weekend differences. For each measure, we required a minimum data availability of 5 days, with at least 3 hours of sleep wear time. Results did not qualitatively change with a minimum data availability of 14 days.

### Statistical Analysis

To evaluate the association between WDSM and LC, we took a cross-sectional approach to compare a 6-month window of wearables data with two consecutive symptom surveys (Fig. 1). For each participant, we selected the first available symptom survey that followed the first availability of the wearables data and was collected at least 6 months post-infection. The subsequent survey timepoint was selected to be the next available survey that was > 1 month and < 6 months following the first one. If no such survey existed in that time window, the second timepoint was determined to be missing. The 6-month window of digital health data was selected such that it was centered around the two survey timepoints. If the second timepoint was missing, the window was selected to begin 45 days before the available symptom survey. Because each wearables measure had different patterns of data availability, this window selection was done independently by measure.

Using the symptom surveys at the two timepoints, we combined the LCRI and LCRS for each participant at each timepoint. The LCRS was taken to be the average value at the two timepoints. A participant was defined as likely LC if their LCRI score was ≥ 11 at either timepoint. For participants with only the first timepoint, we classified them based only on the available data. To examine the implicit assumption “last observation carried forward”, specifically in the LC negative group (LCRI < 11), we performed a sensitivity analysis which excluded these participants. These analyses showed no qualitative differences to the primary analyses.

### Association between sleep measures and long COVID

Logistic regression was used to assess the association between each sleep measure and LCRI or individual symptoms, with the sleep measures being the independent variable. Analogously, associations between sleep measures and continuous measures (LCRS, PROMIS GPH and PROMIS GMH) were assessed using linear regression with the sleep measure as the independent variable.

For each comparison, four different models were considered. Our primary model (model 3) adjusted for known explanatory variables (age, sex, race and ethnicity, and BMI). Additionally, we explored additional variables (alcohol, smoking, seasonality, and time between surveys that may influence LC status:

LCRI/LC Symptom ~ Digital Sleep Measure (1)
LCRI/LC Symptom ~ Digital Sleep Measure + Age + Sex + Race/Ethnicity (2)
LCRI/LC Symptom ~ Digital Sleep Measure + Age + Sex + Race/Ethnicity + BMI (3)
LCRI/LC Symptom ~ Digital Sleep Measure + Age + Sex + Race/Ethnicity + BMI + Alcohol consumption + Smoking + Seasonality + time between surveys + n_records, digital_ (Full)

where n_records, digital_ is the number of Fitbit sleep records in the time window. Because of the small number of participants who are intersex or of unknown sex, these individuals were grouped with the male sex group for the purpose of analysis. To account for multiple significance testing, *p*-values were adjusted using the Benjamini-Hochberg method within LCRI, LCRS and the set of symptoms separately for each model (q-value < 0.05).

### Clustering

Participants were clustered into subgroups using hierarchical clustering based on a subset of digital sleep measures that were strongly correlated with the GPH and GMH variables (for external validity) but were relatively distinct (showing low correlations) from each other (for discriminant validity)^53^. Clusters were generated using the hclust() function in the R “stats” package using the Ward.D2 agglomeration method which minimizes the within-cluster variance. The resulting dendrogram was cut using a hybrid algorithm in the cutreeDynamic() function in the R “dynamicTreeCut” package. The optimal number of clusters was determined based on both the scree plot which shows how the within-cluster sum of squares varies with different numbers of clusters, and the Bayesian Information Criterion (BIC) plot generated using the mclustBIC() function in the R “mclust” package. We further investigated the demographics, presence of sleep-related symptoms, and distribution of quality of life measures within each cluster to identify distinct patterns in subgroups.

### Criterion Profile Analysis

The criterion profile analysis (CPA) was used to determine whether there was a pattern of digital sleep measures associated with the presence of sleep-related symptoms or high scores on quality of life measures^29^. The relative importance of predictors (i.e., the digital sleep measures) was quantified using the criterion pattern score, which was defined as the standardized regression coefficient of a given predictor minus the average of all standardized regression coefficients in the model, multiplied by a positive constant, such that higher absolute criterion pattern scores indicate higher importance in predicting the outcome of interest. Under CPA, the predictor scores are decomposed into two components: a level component (defined as the mean of the predictor scores) and a pattern component (defined as the deviation of each predictor score from the mean predictor score), such that the total variance of the outcome is explained by both the level and the pattern of the predictors. The level and pattern effects can be tested via F-tests by comparing the full model and the model with only level/pattern components. The analysis was done using the cpa() function in the R “profileR” package^54^. Results were generated to show digital sleep measures associated with *presence* of sleep symptoms (e.g., apnea and sleep disturbance) and *decreased* global health scores.

## Supplementary Files

This is a list of supplementary files associated with this preprint. Click to download.

- SleepManuscriptSUpplement20250611.docx

## Figures and Tables

**Table 1:** Wearables Derived Sleep Measures (WDSMs) of Multi-Dimensional Model of Sleep

Dimension	Metric	Description
**Quantity**	Sleep Duration (mins)	Average duration-quantity- of sleep during the main sleep period. Sleep duration is measured as the sum of all periods during the main sleep period spent asleep (vs wake).
**Sleep Depth and Architecture**	Minutes in Light Sleep	Estimated time in stages I and 2 sleep summarized across the entire sleep period.
	Minutes in Deep Sleep	Estimated time in stage 3 (deep) sleep summarized across the entire sleep period.
	Minutes in REM Sleep	Estimated time in Rapid Eye Movement (REM) sleep summarized across the entire sleep period.
**Sleep Quality**	Sleep Efficiency (%)	Percentage time during the overnight sleep (bedtime) period when estimated to be asleep (vs sleep plus wake)
	WASO (Wake After Sleep Onset, minutes)	Total duration in minutes spent awake during the overnight (bedtime) period
	REM fragmentation	Number of times periods of sleep are interrupted by wakefulness
**Sleep Regularity**	Standard Deviation (SD) of Sleep Duration (mins)	The night-to-night regularity in sleep patterns is described by measuring the extent to which sleep duration and sleep timing varies across time periods of 5 of more consecutive days
	SD of Mid-Sleep (mins)	Mid-Sleep is measured as the clock time mid-way between sleep onset and wake time.
**Sleep-Disordered Breathing**	REM Sleep Breathing Rate (per minute)	Estimated rate of breathing during REM sleep.
	SpO2 (%)	Average oxygen saturation during the sleep period.
**Sleep-Related Cardiac Activity**	Heart Rate Variability (HRV; ms)	Beat to beat variation in heart rate during the sleep period.
	Resting Heart Rate (bpm; beats per minute)	Average heart rate during the sleep period.

**Table 2:** Study demographics

Characteristic	Highly Symptomatic LCRS ≥ 11 N = 433^1^	Possible LC 11 > LCRS > 0 N = 529^1^	None LCRS = 0 N = 299^1^	Unknown N = 1^1^
**Sex assigned at birth**				
Male	81 (19%)	136 (26%)	79 (26%)	0 (0%)
Female	351 (81%)	388 (74%)	220 (74%)	1 (100%)
Intersex	0 (0%)	2 (0.4%)	0 (0%)	0 (0%)
Unknown	1	3	0	0
**Age at enrollment**	46 (37, 57)	44 (34, 58)	45 (33, 61)	63 (63, 63)
**Race and ethnicity**				
Asian, Non-Hispanic	13 (3.0%)	31 (5.9%)	33 (11%)	0 (0%)
Black, Non-Hispanic	34 (7.9%)	46 (8.7%)	19 (6.4%)	1 (100%)
Hispanic	56 (13%)	65 (12%)	42 (14%)	0 (0%)
White, Non-Hispanic	304 (70%)	358 (68%)	189 (63%)	0 (0%)
Mixed race/Other/Missing	26 (6.0%)	29 (5.5%)	16 (5.4%)	0 (0%)

1 n (%); Median (Q1, Q3)

**Table 3:** Distribution of wearables derived sleep measures (WDSMs) by Long COVID Research Index (LCRI) group

Characteristic	Long COVID N = 477^1^	Negative Long COVID N = 853^1^
Sleep Efficiency (%)	93.35 (91.01, 94.95)	93.78 (92.08, 95.17)
Sleep Duration (mins)	349.19 (300.27, 391.13)	358.90 (312.96, 399.69)
Minutes in Deep Sleep	60.72 (50.26, 73.45)	63.47 (52.53, 73.37)
Minutes in REM Sleep	82.68 (67.78, 96.71)	83.00 (68.94, 95.36)
REM Sleep Breathing Rate (per minute)	14.19 (12.97, 15.63)	14.51 (13.26, 15.79)
SpO2 (%)	94.75 (93.80, 95.58)	95.07 (94.18, 95.78)
Heart Rate Variability (ms)	22.84 (16.98, 33.02)	27.85 (20.75, 40.78)
Resting Heart Rate (bpm)	68.76 (63.30, 74.20)	66.30 (60.54, 72.09)
SD of Sleep Duration (mins)	112.30 (91.62, 139.09)	98.50 (75.75, 123.03)
SD of Mid-Sleep (mins)	24.12 (17.58, 34.07)	19.79 (13.91, 29.19)
REM Onset Latency (mins)	119.50 (99.04, 141.67)	110.80 (94.33, 131.61)
REM Fragmentation Index	3.33 (2.92, 3.99)	3.38 (3.00, 3.96)
Minutes in Light Sleep	248.81 (218.90, 275.71)	247.48 (221.20, 272.90)
WASO (mins)	47.22 (38.54, 55.10)	48.86 (40.59, 57.11)

1 Median (Q1, Q3)

**Table 4:** Demographic and symptom characteristics by cluster

Characteristic	Cluster 1 N = 459^1^	Cluster 2 N = 266^1^	Cluster 3 N = 253^1^	Cluster 4 N = 150^1^
**LCRI Category**				
Positive	160 (35%)	54 (20%)	104 (41%)	72 (48%)
Negative	299 (65%)	212 (80%)	149 (59%)	78 (52%)
**Sex assigned at birth**				
Male	96 (21%)	74 (28%)	69 (27%)	24 (16%)
Female	361 (79%)	190 (72%)	184 (73%)	124 (84%)
Intersex	1 (0.2%)	1 (0.4%)	0 (0%)	0 (0%)
Unknown	1	1	0	2
**Age at enrollment**	48 (36, 60)	36 (30, 48)	49 (38, 60)	47 (38, 57)
**Race and ethnicity**				
Non-Hispanic Asian	18 (3.9%)	29 (11%)	22 (8.7%)	3 (2.0%)
Non-Hispanic Black	19 (4.1%)	22 (8.3%)	37 (15%)	9 (6.0%)
Hispanic	58 (13%)	35 (13%)	30 (12%)	21 (14%)
Non-Hispanic White	345 (75%)	160 (60%)	145 (57%)	110 (73%)
Mixed race/Other/Missing	19 (4.1%)	20 (7.5%)	19 (7.5%)	7 (4.7%)
**Sleep Apnea**	115 (25%)	35 (13%)	94 (38%)	54 (37%)
Unknown	4	4	3	4
**Sleep Disturbance**	63 (14%)	24 (9.2%)	54 (22%)	41 (28%)
Unknown	4	4	3	4
**PROMIS Global Physical (t-score)**	49 (42, 54)	52 (46, 58)	46 (39, 52)	45 (39, 51)
**PROMIS Global Mental (t-score)**	47 (40, 53)	50 (44, 55)	45 (39, 51)	45 (39, 52)

1 n (%); Median (Q1, Q3)

## Data Availability

The RECOVER Adult Cohort Observational Study remains in progress and therefore datasets are updated dynamically, individual participant-level questionnaire-based data including the data dictionary is available on the RECOVER website (https://recovercovid.org/). NHLBI has undertaken a significant effort to release harmonized data from all RECOVER observational cohort studies to the public via their BioData Catalyst platform (https://biodatacatalyst.nhlbi.nih.gov/). Datasets include participant-level data collected for the present study, as well as the technical infrastructure and analytic tools to evaluate the results and conclusions from this study.

## References

[1] Ford N. D. Notes from the Field: Long COVID Prevalence Among Adults - United States, 2022. MMWR Morb Mortal Wkly Rep 73, 135–136 (2024). 10.15585/mmwr.mm7306a4 38359012 PMC10899083

[2] Cutler D. M. The Costs of Long COVID. JAMA Health Forum 3, e221809 (2022). 10.1001/jamahealthforum.2022.1809 36219031

[3] 2025 Long COVID Fact Sheet, <https://patientresearchcovid19.com/2025-long-covid-fact-sheet/> (2025).

[4] Cutler D. M. The Economic Cost of Long COVID: An Update, 2025).

[5] Tsilingiris D. Laboratory Findings and Biomarkers in Long COVID: What Do We Know So Far? Insights into Epidemiology, Pathogenesis, Therapeutic Perspectives and Challenges. Int J Mol Sci 24 (2023). 10.3390/ijms241310458 PMC10341908 37445634

[6] National Center for Health Statistics. U.S. Census Bureau, Household Pulse Survey, 2022–2024. Long COVID., <https://www.cdc.gov/nchs/covid19/pulse/long-covid.htm> (2025).

[7] Erlandson K. M. Differentiation of Prior SARS-CoV-2 Infection and Postacute Sequelae by Standard Clinical Laboratory Measurements in the RECOVER Cohort. Ann Intern Med 177, 1209–1221 (2024). 10.7326/M24-0737 39133923 PMC11408082

[8] National Academies of Sciences, Engineering, and Medicine; Health and Medicine Division; Board on Global Health; Board on Health Sciences Policy; Committee on Examining the Working Definition for Long COVID. (National Academies Press (US), 2024).

[9] Perumal R. Long COVID: a review and proposed visualization of the complexity of long COVID. Front Immunol 14, 1117464 (2023). 10.3389/fimmu.2023.1117464 37153597 PMC10157068

[10] Pena-Orbea C. Sleep Disturbance Severity and Correlates in Post-acute Sequelae of COVID-19 (PASC). J Gen Intern Med 38, 2015–2017 (2023). 10.1007/s11606-023-08187-3 37014604 PMC10072019

[11] Tedjasukmana R., Budikayanti A., Islamiyah W. R., Witjaksono A. & Hakim M. Sleep disturbance in post COVID-19 conditions: Prevalence and quality of life. Front Neurol 13, 1095606 (2022). 10.3389/fneur.2022.1095606 36698905 PMC9869804

[12] Garbarino S., Lanteri P., Bragazzi N. L., Magnavita N. & Scoditti E. Role of sleep deprivation in immune-related disease risk and outcomes. Commun Biol 4, 1304 (2021). 10.1038/s42003-021-02825-4 34795404 PMC8602722

[13] Simon K. C., Nadel L. & Payne J. D. The functions of sleep: A cognitive neuroscience perspective. Proc Natl Acad Sci U S A 119, e2201795119 (2022). 10.1073/pnas.2201795119 PMC9636951 36279445

[14] Xie L. Sleep drives metabolite clearance from the adult brain. Science 342, 373–377 (2013). 10.1126/science.1241224 24136970 PMC3880190

[15] M. H, L. Risk of post-acute sequelae of SARS-CoV-2 infection associated with pre-coronavirus disease obstructive sleep apnea diagnoses: an electronic health record-based analysis from the RECOVER initiative. Sleep 46 (2023). 10.1093/sleep/zsad126 PMC10485569 37166330

[16] Schilling C. Pre-existing sleep problems as a predictor of post-acute sequelae of COVID-19. J Sleep Res 33, e13949 (2024). 10.1111/jsr.13949 37227000

[17] Quan S. F. Sleep and long COVID: preexisting sleep issues and the risk of post-acute sequelae of SARS-CoV-2 infection in a large general population using 3 different model definitions. J Clin Sleep Med 21, 249–259 (2025). 10.5664/jcsm.11322 39324686 PMC11789238

[18] de Zambotti M., Cellini N., Goldstone A., Colrain I. M. & Baker F. C. Wearable Sleep Technology in Clinical and Research Settings. Med Sci Sports Exerc 51, 1538–1557 (2019). 10.1249/MSS.0000000000001947 30789439 PMC6579636

[19] Phillips A. J. K. Irregular sleep/wake patterns are associated with poorer academic performance and delayed circadian and sleep/wake timing. Sci Rep 7, 3216 (2017). 10.1038/s41598-017-03171-4 28607474 PMC5468315

[20] Lee T. Accuracy of 11 Wearable, Nearable, and Airable Consumer Sleep Trackers: Prospective Multicenter Validation Study. JMIR Mhealth Uhealth 11, e50983 (2023). 10.2196/50983 37917155 PMC10654909

[21] Berryhill S. Effect of wearables on sleep in healthy individuals: a randomized crossover trial and validation study. J Clin Sleep Med 16, 775–783 (2020). 10.5664/jcsm.8356 32043961 PMC7849816

[22] Geng L. N. 2024 Update of the RECOVER-Adult Long COVID Research Index. JAMA (2024). 10.1001/jama.2024.24184 PMC11862971 39693079

[23] Haghayegh S., Khoshnevis S., Smolensky M. H., Diller K. R. & Castriotta R. J. Accuracy of Wristband Fitbit Models in Assessing Sleep: Systematic Review and Meta-Analysis. J Med Internet Res 21, e16273 (2019). 10.2196/16273 31778122 PMC6908975

[24] Radin J. M. Long-term changes in wearable sensor data in people with and without Long Covid. NPJ Digit Med 7, 246 (2024). 10.1038/s41746-024-01238-x 39271927 PMC11399345

[25] Kukreti S., Lu M. T., Yeh C. Y. & Ko N. Y. Physiological Sensors Equipped in Wearable Devices for Management of Long COVID Persisting Symptoms: Scoping Review. J Med Internet Res 27, e69506 (2025). 10.2196/69506 40137051 PMC11982746

[26] Mekhael M. Studying the Effect of Long COVID-19 Infection on Sleep Quality Using Wearable Health Devices: Observational Study. J Med Internet Res 24, e38000 (2022). 10.2196/38000 35731968 PMC9258734

[27] Thaweethai T. Development of a Definition of Postacute Sequelae of SARS-CoV-2 Infection. JAMA 329, 1934–1946 (2023). 10.1001/jama.2023.8823 37278994 PMC10214179

[28] Hays R. D., Bjorner J. B., Revicki D. A., Spritzer K. L. & Cella D. Development of physical and mental health summary scores from the patient-reported outcomes measurement information system (PROMIS) global items. Qual Life Res 18, 873–880 (2009). 10.1007/s11136-009-9496-9 19543809 PMC2724630

[29] Davison M. L. & Davenport E. C. Jr. Identifying criterion-related patterns of predictor scores using multiple regression. Psychol Methods 7, 468–484 (2002). 10.1037/1082-989x.7.4.468 12530704

[30] Haischer M. H. Heart rate variability is reduced in COVID-19 survivors and associated with physical activity and fatigue. Physiol Rep 12, e15912 (2024). 10.14814/phy2.15912 38243329 PMC10799199

[31] da Silva A. L. G. Impact of long COVID on the heart rate variability at rest and during deep breathing maneuver. Sci Rep 13, 22695 (2023). 10.1038/s41598-023-50276-0 38123689 PMC10733257

[32] Georgiou K. Can Wearable Devices Accurately Measure Heart Rate Variability? A Systematic Review. Folia Med (Plovdiv) 60, 7–20 (2018). 10.2478/folmed-2018-0012 29668452

[33] Sanches C. A., Silva G. A., Librantz A. F. H., Sampaio L. M. M. & Belan P. A. Wearable Devices to Diagnose and Monitor the Progression of COVID-19 Through Heart Rate Variability Measurement: Systematic Review and Meta-Analysis. J Med Internet Res 25, e47112 (2023). 10.2196/47112 37820372 PMC10685286

[34] Mauro M., Cegolon L., Bestiaco N., Zulian E. & Larese Filon F. Heart Rate Variability Modulation Through Slow-Paced Breathing in Health Care Workers with Long COVID: A Case-Control Study. Am J Med 138, 870–883.e875 (2025). 10.1016/j.amjmed.2024.05.021 38795941

[35] Leproult R., Holmback U. & Van Cauter E. Circadian misalignment augments markers of insulin resistance and inflammation, independently of sleep loss. Diabetes 63, 1860–1869 (2014). 10.2337/db13-1546 24458353 PMC4030107

[36] Huang T., Mariani S. & Redline S. Sleep Irregularity and Risk of Cardiovascular Events: The Multi-Ethnic Study of Atherosclerosis. J Am Coll Cardiol 75, 991–999 (2020). 10.1016/j.jacc.2019.12.054 32138974 PMC7237955

[37] Tracy E. L. Multidimensional Sleep and Self-Rated Physical Health and Depressive Symptoms Among Retired Older Adults: A Sex-Stratified Analysis. Behav Sleep Med, 1–12 (2025). 10.1080/15402002.2025.2493651 PMC12213196 40243097

[38] Hilser J. R. COVID-19 Is a Coronary Artery Disease Risk Equivalent and Exhibits a Genetic Interaction With ABO Blood Type. Arterioscler Thromb Vasc Biol 44, 2321–2333 (2024). 10.1161/atvbaha.124.321001 39381876 PMC11495539

[39] Coelho F. M. S. Sleep disorder syndromes of post-acute sequelae of SARS-CoV-2 (PASC) / Long Covid. Sleep Med 123, 37–41 (2024). 10.1016/j.sleep.2024.08.030 39236463

[40] Tobin M. J., Laghi F. & Jubran A. Why COVID-19 Silent Hypoxemia Is Baffling to Physicians. Am J Respir Crit Care Med 202, 356–360 (2020). 10.1164/rccm.202006-2157CP 32539537 PMC7397783

[41] Halawa S. Potential long-term effects of SARS-CoV-2 infection on the pulmonary vasculature: a global perspective. Nat Rev Cardiol 19, 314–331 (2022). 10.1038/s41569-021-00640-2 34873286 PMC8647069

[42] Sun H. Facility-Measured Nocturnal Hypoxemia and Sleep Among Adults with Long COVID versus Age- and Sex-Matched Healthy Adults: A Preliminary Observational Study. SLEEP Advances (2025). 10.1093/sleepadvances/zpaf017 PMC12070477 40365527

[43] Subramanian A. Symptoms and risk factors for long COVID in non-hospitalized adults. Nat Med 28, 1706–1714 (2022). 10.1038/s41591-022-01909-w 35879616 PMC9388369

[44] Kendzerska T., Leung R. S., Gershon A. S., Tomlinson G. & Ayas N. The Interaction of Obesity and Nocturnal Hypoxemia on Cardiovascular Consequences in Adults with Suspected Obstructive Sleep Apnea. A Historical Observational Study. Ann Am Thorac Soc 13, 2234–2241 (2016). 10.1513/AnnalsATS.201604-263OC 27690206

[45] Gabbay I. E., Gabbay U. & Lavie P. Obesity plays an independent worsening modifying effect on nocturnal hypoxia in obstructive sleep apnea. Sleep Med 13, 524–528 (2012). 10.1016/j.sleep.2012.01.005 22349363

[46] Guerra S. Morbidity and mortality associated with the restrictive spirometric pattern: a longitudinal study. Thorax 65, 499–504 (2010). 10.1136/thx.2009.126052 20522846 PMC3036842

[47] Bonsignore M. R. REM sleep obstructive sleep apnoea. Eur Respir Rev 33 (2024). 10.1183/16000617.0166-2023 PMC10865098 38355150

[48] Rouen A. Polysomnographic parameters in long-COVID chronic insomnia patients. Dialogues Clin Neurosci 25, 43–49 (2023). 10.1080/19585969.2023.2222714 37390849 PMC10316734

[49] Sramek J. The effect of naltrexone on sleep parameters in healthy male volunteers. J Clin Psychopharmacol 34, 167–168 (2014). 10.1097/JCP.0b013e3182a607ff 24346745

[50] Jin J. Association of rapid eye movement sleep latency with multimodal biomarkers of Alzheimer’s disease. Alzheimers Dement 21, e14495 (2025). 10.1002/alz.14495 39868572 PMC11848184

[51] Horwitz L. I. Researching COVID to Enhance Recovery (RECOVER) adult study protocol: Rationale, objectives, and design. PLoS One 18, e0286297 (2023). 10.1371/journal.pone.0286297 37352211 PMC10289397

[52] von Gall C., Holub L., Ali A. A. H. & Eickhoff S. Timing of Deep and REM Sleep Based on Fitbit Sleep Staging in Young Healthy Adults under Real-Life Conditions. Brain Sci 14 (2024). 10.3390/brainsci14030260 PMC10968898 38539648

[53] Little T., Lindenberger U. & Nesselroade J. R. On selecting indicators for multivariate measurement and modeling with latent variables. Psychological Methods 4 (1999).

[54] Desjardins C. D. & Bulut O. profileR: An R package for profile analysis. Journal of Open Source Software 5, 1941 (2020). 10.21105/joss.01941

